# Supplementary material for: Heat Acclimatization, Cooling Strategies, and Hydration during an Ultra-Trail in Warm and Humid Conditions
Source: Nutrients. 2021 Mar 26;13(4):1085. doi: 10.3390/nu13041085 (PMC8065615; doi:10.3390/nu13041085)
Supplement: Supplementary file 1 [file nutrients-13-01085-s001.pdf]

Article

Heat Acclimatization, Cooling Strategies and Hydration during an Ultra-Trail in Warm and Humid Conditions

Nicolas Bouscaren [1,2,*], Robin Faricier [2], Guillaume Y Millet [2,3,$], Sébastien Racinais [4,$]

1 Inserm CIC1410, CHU Réunion, Saint Pierre, La Réunion, France ; nicolas.bouscaren@chu-reunion.fr
2 Univ Lyon, UJM-Saint-Etienne, Inter-university Laboratory of Human Movement Biology, EA 7424, F-42023, Saint-Etienne, France ; robinfaricier@live.fr ;
3 guillaume.millet@univ-st-etienne.fr
4 Institut Universitaire de France (IUF)
5 Research and Scientific Support Department, Aspetar Orthopedic and Sports Medicine Hospital, Doha, Qatar ; Sebastien.Racinais@aspetar.com

* Correspondence: nicolas.bouscaren@chu-reunion.fr ; +262 262 71 98 30
$ These two authors contributed equally to the manuscript.

Citation: Bouscaren, N.; Faricier, R.; Millet, G.Y.; Racinais, N. Hydration, heat acclimatization, and cooling strategies for ultra-trail in warm and humid environment. *Nutrients* **2021**, *13*, 1085. https://doi.org/10.3390/nu13041085

Received: 17 February 2021
Accepted: 24 March 2021
Published: 26 March 2021

Publisher's Note: MDPI stays neutral with regard to jurisdictional claims in published maps and institutional affiliations.

Copyright: © 2020 by the authors. Submitted for possible open access publication under the terms and conditions of the Creative Commons Attribution (CC BY) license (http://creativecommons.org/licenses/by/4.0/).

Suplementary material

Supplementary S1: Pre-race survey (French version)

Supplementary S2: Post-race survey (French version)

Finalité de l'étude

» Objectif : décrire la préparation des coureurs de trail en vue de la participation à une épreuve se déroulant en environnement chaud et humide

» Public visé : coureurs participants à l'une des 3 courses individuelles du Grand Raid Réunion : La Mascareignes, Le Trail de Bourbon et La Diagonale des Fous

Vos droits

Un traitement informatique de vos données personnelles va être mis en œuvre pour permettre d'analyser les résultats de la recherche :

» Vous disposez à tout moment d'un droit d'accès à vos données. Vous pouvez les faire rectifier, les effacer, vous opposer ou en limiter le traitement.

» Vos données seront conservées le temps de la publication d'un article scientifique en lien avec l'utilisation de ces données.

» Lorsque cette recherche sera terminée, vous serez tenus informés directement par mail et sur le site du Grand Raid Réunion des résultats de l'étude

Contact

Si vous avez des questions ou des retours éventuels, merci de contacter :
Dr. Nicolas BOUSCAREN – Responsable de la recherche
0692 78 30 32
nicolas.bouscaren@chu-reunion.fr

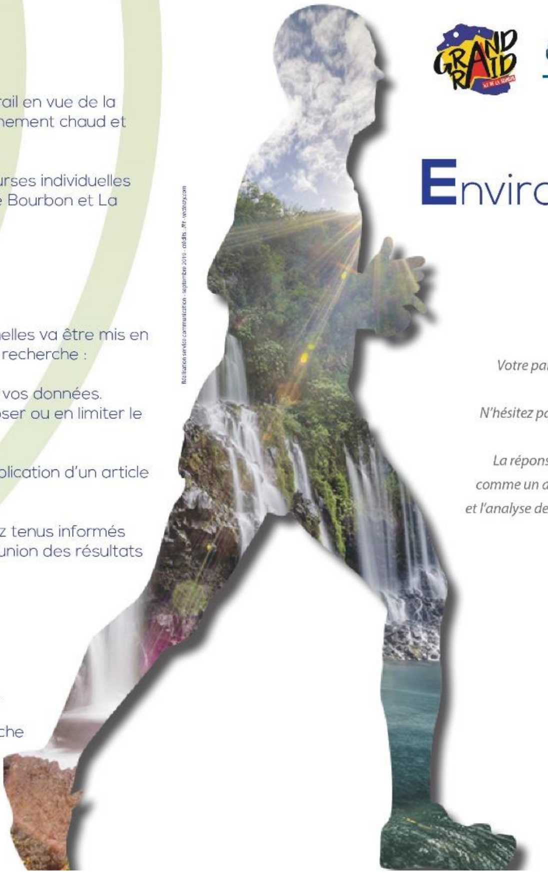

QUESTIONNAIRE

Trail & Environnement Chaud

Madame, Monsieur,
Votre participation est entièrement libre et volontaire.
N'hésitez pas à poser toutes les questions que vous jugerez utiles.
La réponse au questionnaire est considérée comme un accord de votre part pour le recueil et l'analyse de vos données personnelles dans le cadre de cette recherche.

CIC Réunion – Centre d'investigation clinique
CHU – Centre Hospitalier Universitaire – La Réunion

I - INFORMATIONS GÉNÉRALES

1. Sexe : ☐ M / ☐ F
2. Année de naissance : _______
3. Poids : ______kg **4. Taille :** ______cm
5. Votre résidence principale est-elle à La Réunion ?
☐ Oui ☐ Non
Si non, date d'arrivée à La Réunion : ____/____/____
Arrivant de _______________________
6. Sur quelle course êtes-vous inscrit(e) cette année ?
☐ La Diagonale des Fous
☐ Le Trail de Bourbon
☐ La Mascareignes
7. Quel est votre numéro de dossard : ________

II - EXPÉRIENCE EN TRAIL

8. Depuis combien d'années pratiquez-vous le trail ? ____ ans

9. Avant cette course, à combien de trail de plus de 60 km avez-vous participé dans votre vie ? _____ trail(s)

10. Concernant votre entraînement
a) En moyenne **lors des 6 derniers mois,** quel a été votre **nombre d'heure d'entraînement hebdomadaire** ? (tout type d'entraînement confondu) _____ heures/semaine
b) En moyenne **lors des 6 derniers mois,** quel a été le **nombre de kilomètre hebdomadaire parcouru en course à pied/trail** ?
______ kilomètres/semaine
c) En moyenne **lors des 6 derniers mois**, quel a été le **dénivelé positif hebdomadaire en course à pied/trail** ?
______ mètres D+/semaine

III - PROBLÈMES DE SANTÉ AVANT LE DÉPART

11. Avez-vous éprouvé un ou plusieurs des symptômes suivants au cours des 4 semaines précédant cette course ?
☐ Fièvre
☐ Congestion, nez bouché
☐ Éternuements
☐ Vomissements
☐ Douleurs abdominales
☐ Diarrhée
☐ Céphalées/maux de tête
☐ Pathologie en lien avec la chaleur
☐ Fatigue générale
☐ Autre : (décrivez les SVP)
....
....
☐ Aucun symptôme

IV - EXPÉRIENCE EN TRAIL EN ENVIRONNEMENT CHAUD

Dans cette rubrique, vous pourrez décrire vos expériences en lien avec la pratique du trail et/ou de la course à pied en environnement chaud.

13. Avez-vous déjà ressenti les signes ou symptômes suivant que vous attribuez à une course ou un entraînement en environnement chaud ? (plusieurs réponses possibles)
☐ Crampes
☐ Nausées
☐ Vomissements
☐ Maux de tête sévères
☐ Malaise/évanouissement
☐ Fatigue/diminution des performances
☐ Autres ....
☐ Aucun de ces signes, je n'ai jamais souffert de la chaleur

14. Avez-vous déjà été diagnostiqué / traité par un médecin pour un problème lié à l'exercice en environnement chaud ?
☐ Oui ☐ Non
Si non vous pouvez passer à la question 18 directement.

15. Si oui, s'agissait-il de :
☐ Déshydratation
☐ Hyponatrémie
☐ Épuisement dû à la chaleur
☐ Coup de chaleur
☐ Autre (précisez) ....

16. Êtes-vous allé aux urgences ou avez-vous été hospitalisé du fait de cette pathologie ?
☐ Oui ☐ Non

17. Si oui, étiez-vous hospitalisé en réanimation ?
☐ Oui ☐ Non

18. Pour les non-résidents à La Réunion, spécifiquement pour la préparation de cette course avant votre arrivée sur l'Île, vous êtes-vous (plusieurs réponses possibles) :
☐ Spécifiquement entraîné en milieu chaud naturel
Combien de jours ? ______
☐ Spécifiquement entraîné en chambre artificielle chaude/laboratoire
Combien de jours ? ______
☐ Je ne me suis pas entraîné spécifiquement en environnement chaud
☐ J'habite dans un pays avec un climat tropical ou désertique

19. Pour les non-résidents à La Réunion, avez-vous volontairement programmé une arrivée précoce sur l'île dans le but de vous acclimater aux conditions environnementales locales (hors contexte de vacances) **?**
☐ Oui ☐ Non

20. Pour les non-résidents à La Réunion, le plus souvent, vous vous entraînez dans un milieu correspondant à quel type de climat ?
☐ Climat tempéré (océanique, continental ou méditerranéen)
☐ Climat tropical ou équatorial
☐ Climat désertique
☐ Climat subarctique ou polaire

V - VOTRE STRATÉGIE DE COURSE

21. Concernant votre tenue pendant la course
a) Utilisez-vous des manchons/chaussettes/bas de compression au niveau des mollets ? ☐ Oui ☐ Non
b) Utilisez-vous un cuissard de compression au niveau des cuisses ?
☐ Oui ☐ Non
c) Avez-vous choisi la couleur de votre tenue en vue de limiter l'absorption solaire (tenue de couleur claire) ?
☐ Oui ☐ Non

22. Quelle quantité de liquide prévoyez-vous de consommer en moyenne pendant votre course ? _______ millilitres/heure

23. Quelle(s) type(s) de boisson(s) comptez-vous utiliser ? (plusieurs réponses possibles, indiquez le pourcentage approximatif en fonction du volume total de boissons pour chaque case cochée)
☐ Eau pure ____%
☐ Eau enrichie en sel ____%
☐ Boisson avec préparation maison ____%
Composition : ....
☐ Boisson de l'effort vendue dans le commerce ____%
Marque : ....
☐ Autre boisson (coca, Red Bull, etc.) ____%
Marque ou type de boisson : ....

24. Quelle stratégie de rafraîchissement prévoyez-vous de mettre en place après la course ? (plusieurs réponses possibles)
☐ Serviette froide
☐ Immersion corps entier dans l'eau froide
☐ Immersion des jambes dans l'eau froide
☐ Ingestion d'eau glacée
☐ Éponge mouillée
☐ Arrêt/repos à l'ombre
☐ Gilet réfrigérant
☐ Tour de cou réfrigérant
☐ Autre (précisez) ....
☐ Je n'ai pas prévu de mettre en place de stratégie de rafraîchissement

25. Quelle stratégie de récupération prévoyez-vous de mettre en place après la course ? (plusieurs réponses possibles)
☐ Récupération active
☐ Étirement
☐ Massage
☐ Immersion dans l'eau froide
☐ Immersion dans l'eau chaude, sauna, hammam
☐ Électrostimulation
☐ Immersion chaud/froid alterné, douche écossaise
☐ Chaussettes, collants de compression
☐ Autre (précisez) ....
☐ Je n'ai pas prévu de mettre en place de stratégie de récupération

Supplementary S1: Pre-race survey (French version)

Supplementary S2: Post-race survey (French version)

Information générale

1. A quelle course avez-vous participé(e) cette année ?

☐ La Diagonale des Fous

☐ Le Trail de Bourbon

☐ La Mascareignes

2. Quel était votre numéro de dossard : _ _ _ _

Si vous aviez rempli le questionnaire « avant course » concernant votre « préparation » vous pouvez directement passer à la section 3. « Trail et environnement chaud, votre vécu de la course »

3. Sexe : ☐ M / ☐ F **4. Date de naissance (mois/année) :** _ _ / _ _ _ _

5. Poids : _ _ _ _ kg **6. Taille :** _ _ _ _ cm

7. Votre résidence principale est-elle à la Réunion ? ☐ Oui ☐ Non

Si non, date d'arrivée à la Réunion : _ _ / _ _ / _ _ _ _

Arrivant de : Pays

Expérience en Trail

8. Depuis combien d'années pratiquez-vous le Trail ?

(Si vous avez débuté cette année renseignez 1)

_ _ ans

9. Avant cette course, à combien de trail de plus de 60 km avez-vous participé dans votre vie ?

_ _ trail(s)

10. Concernant votre entrainement

a) En moyenne **lors des 6 derniers mois**, quel a été votre **nombre d'heure d'entrainement hebdomadaire** pour la préparation de cette course (tout type d'entrainement confondu :renforcements musculaires, course à pied, préparation physique…) **?**

En moyenne : _ _ heures/semaine

b) En moyenne **lors des 6 derniers mois**, quelle a été **le nombre de kilomètre hebdomadaire parcouru en course à pied/trail** pour la préparation de cette course ?

En moyenne : _ _ _ kilomètres/semaine

c) En moyenne **lors des 6 derniers mois,** quel a été **le dénivelé positif hebdomadaire en course à pied/trail** pour la préparation de cette course ?

En moyenne : _ _ _ _ mètres D+/semaine

Trail et environnement chaud : votre vécu de la course

11. Avez-vous souffert de la chaleur durant votre course ?

☐ Oui ☐ Non

12. Selon vous, votre performance a-t ’elle était impactée par la chaleur au cours de cette course ?

☐ Oui de manière négative

☐ Oui de manière négative

☐ Non, la chaleur n’a en rien impacté ma performance

14. Êtes-vous « finisher » de la course à laquelle vous avez participée ?

☐ Oui ☐ Non

Si oui passez à la question 18 directement

15. Si non attribuez-vous, au moins en partie, votre abandon aux conditions environnementales chaudes sur la course ou à des problèmes de régulation de température de votre organisme ?

☐ Oui ☐ Non

16. Sur cette course, êtes-vous allé aux urgences ou avez-vous été hospitalisé du fait de la survenue d’une pathologie en lien avec la chaleur ?

☐ Oui ☐ Non

Si non vous pouvez passer à la question 18 directement.

17. Si oui, s’agissait-il de :

☐ Coup de chaleur

☐ Autre (précisez SVP)

……………………………………..

☐ Déshydratation

☐ Hyponatrémie

☐ Épuisement dû à la chaleur

18. Durant votre course, avez-vous ressenti les symptômes suivants, dont l’origine serait d’après vous, en lien avec la chaleur ou une mauvaise gestion de votre hydratation (Plusieurs réponses possibles)?

☐ Vomissements

☐ Maux de tête sévères

☐ Crampes

☐ Nausées

……………………………………..

☐ Aucun de ces signes, je n'ai pas souffert de la chaleur

☐ Malaise/évanouissement

☐ Fatigue/diminution des performances

☐ Autre (précisez)

19. Concernant votre hydratation, quelle quantité de liquide avez-vous consommé en moyenne pendant votre course ?

En moyenne : _ _ _ millilitres/heure

20. Concernant votre hydratation quels facteurs ont guidé votre consommation de boissons pendant la course

☐ La soif, les muqueuses sèches

☐ Les urines foncées

☐ Rythme de consommation planifié, stratégie de consommation

☐ Le maximum toléré

21. Selon vous, votre hydratation a-t-elle était suffisante sur cette course ?

☐ Oui ☐ Non

22. Quelle(s) type(s) de boisson(s) avez-vous utilisé (Plusieurs réponses possibles) ?

☐ Eau pure

☐ Eau enrichie en sel

☐ Boisson avec préparation maison

Composition :

☐ Boisson de l'effort vendu dans le commerce

Marque :

☐ Autre boisson (coca, Red bull, etc.)

Marque ou type de boisson :

23. Avez-vous consommé du sel ou enrichie vos boissons ou vos aliments en sel ?

☐ Oui ☐ Non

24. Durant la course Avez-vous utilisé des stratégies de rafraîchissement (=percooling) ?

☐ Oui ☐ Non

25. Si oui la ou lesquelles (plusieurs réponses possibles) ?

☐ Arrêt/repos à l'ombre

☐ Gilet réfrigérant

☐ Tour de cou réfrigérant

☐ Autre (précisez)

☐ Serviette froide

☐ Immersion corps entier dans l'eau froide

☐ Immersion des jambes dans l'eau froide

☐ Ingestion d'eau glacée

☐ Eponge mouillée

26. Quelle stratégie de récupération prévoyez-vous de mettre en place (plusieurs réponses possibles)?

☐ Aucune

☐ Récupération active

☐ Etirement

☐ Massage

☐ Immersion dans l'eau froide

☐ Immersion dans l'eau chaude

☐ Immersion chaud/froid alterné, douche écossaise

☐ Electrostimulation

☐ Vêtement de compression

☐ Autre (précisez) :

27. Votre expérience acquise au cours de cette course en lien avec les problématiques de chaleur va t'elle entrainer des modifications pour vos courses futures? (Plusieurs réponses sont possibles)

☐ Oui, je planifierai une arrivée plus précoce pour me permettre de m'acclimater aux conditions environnementales locales

☐ Oui dans ma préparation, je m'entrainerai plus spécifiquement en milieu chaud naturel

☐ Oui pendant ma course, je mettrai en place ou modifierai mes stratégies de rafraichissement (immersion dans l'eau froide, utilisation de serviette froide, ingestion d'eau glacée …)

☐ Oui pendant ma course, je modifierai mes stratégies d'hydratation

- ☐ J'augmenterai le volume d'eau que j'ingère
- ☐ Je diminuerai le volume d'eau que j'ingère
- ☐ Je modifierai le type de boisson que j'utilise

☐ Autre (précisez) :

☐ Non car je suis satisfait de la manière dont s'est déroulé ma course concernant les problématiques de chaleur et d'hydratation

28. Pour les non-résidents à La Réunion à quelle date est prévue/s'est déroulé votre vol retour ?

_ _ / _ _ / _ _ _
